# EEG-Based Eye Movement Recognition Using Brain–Computer Interface and Random Forests

**DOI:** 10.3390/s21072339

**Published:** 2021-03-27

**Authors:** Evangelos Antoniou, Pavlos Bozios, Vasileios Christou, Katerina D. Tzimourta, Konstantinos Kalafatakis, Markos G. Tsipouras, Nikolaos Giannakeas, Alexandros T. Tzallas

**Affiliations:** 1 Department of Informatics and Telecommunications, University of Ioannina, GR47100 Arta, Greece; vaggelisant7@gmail.com (E.A.); boziospavlos@gmail.com (P.B.); bchristou1@gmail.com (V.C.); ktzimourta@uoi.gr (K.D.T.); k.kalafatakis@uoi.gr (K.K.); giannakeas@uoi.gr (N.G.); 2 Q Base R&D, Science & Technology Park of Epirus, University of Ioannina Campus, GR45110 Ioannina, Greece; 3 Department of Electrical and Computer Engineering, University of Western Macedonia, GR50100 Kozani, Greece; mtsipouras@uowm.gr

**Keywords:** brain–computer interface, electroencephalogram, electrooculogram, eye tracking, random forests, EEG, eye movement, EPOC Flex

## Abstract

Discrimination of eye movements and visual states is a flourishing field of research and there is an urgent need for non-manual EEG-based wheelchair control and navigation systems. This paper presents a novel system that utilizes a brain–computer interface (BCI) to capture electroencephalographic (EEG) signals from human subjects while eye movement and subsequently classify them into six categories by applying a random forests (RF) classification algorithm. RF is an ensemble learning method that constructs a series of decision trees where each tree gives a class prediction, and the class with the highest number of class predictions becomes the model’s prediction. The categories of the proposed random forests brain–computer interface (RF-BCI) are defined according to the position of the subject’s eyes: open, closed, left, right, up, and down. The purpose of RF-BCI is to be utilized as an EEG-based control system for driving an electromechanical wheelchair (rehabilitation device). The proposed approach has been tested using a dataset containing 219 records taken from 10 different patients. The BCI implemented the EPOC Flex head cap system, which includes 32 saline felt sensors for capturing the subjects’ EEG signals. Each sensor caught four different brain waves (delta, theta, alpha, and beta) per second. Then, these signals were split in 4-second windows resulting in 512 samples per record and the band energy was extracted for each EEG rhythm. The proposed system was compared with naïve Bayes, Bayes Network, k-nearest neighbors (K-NN), multilayer perceptron (MLP), support vector machine (SVM), J48-C4.5 decision tree, and Bagging classification algorithms. The experimental results showed that the RF algorithm outperformed compared to the other approaches and high levels of accuracy (85.39%) for a 6-class classification are obtained. This method exploits high spatial information acquired from the Emotiv EPOC Flex wearable EEG recording device and examines successfully the potential of this device to be used for BCI wheelchair technology.

## 1. Introduction

Wheelchairs are indispensable aids for individuals with significant mobility impairment, assisting them in daily life routine tasks. The interaction between the patients’ intentions and wheelchair control commands is currently realized using a manual human–machine interface (HMI) with tools like joysticks and keyboards. Although these manual HMIs are very useful, they are ineffective in severe paralysis cases. These cases including the late stages of amyotrophic lateral sclerosis (ALS) and quadriplegia. Such patients can seriously benefit from using a wheelchair controlled by non-manual means [1,2]. Designing a non-manual HMI is challenging because it must allow the patient to control the wheelchair accurately. Since many patients with severe paralysis retain intact brain function, head, and eye movements, a significant number of approaches focus on methods based on brain activity. Such methods utilize electroencephalography/EEG combined with eye movements using electrooculography/EOG for the wheelchair movement [1,3].

Many scientific reports focus on EEG signal processing for the creation of a control mechanism or for emotion recognition. Itturate et al. [4] proposed a neural dynamics-controlled wheelchair based on the EEG signal, which uses the P300 neurophysiological protocol. The system utilizes a BCI connected to the wheelchair where the patient sees a virtual reconstruction of the environment (using the sensors of the wheelchair) and focuses on the area he wants to reach. A visual stimulus extracts the neurological event, and the EEG signal processing software detects the patient’s target location area. The target location area is an area given to the autonomous navigation system, which drives the wheelchair to avoid any objects blocking the way. Huang et al. [5] proposed a 2D virtual wheelchair control system based on event-related desynchronization/synchronization and state control. Craig et al. [6] presented a real-time EEG classification system for a brain-controlled wheelchair used by patients suffering from chronic spinal cord injury (SCI). Mirza et al. [7] utilized a brain-controlled wheelchair using EEG signal processing. The captured EEG signals are translated into moving commands using an Arduino microcontroller. Zgallai et al. [8] presented a 3D printed smart wheelchair prototype using the Emotiv EPOC headset for blind and paralyzed people. This prototype uses deep learning to recognize four different movements (left, right, forward, and stop) taken from the EEG signal, which was captured from the headset. Paul and Moumita [9] proposed a BCI technique based on EEG signal processing for non-manual wheelchair control. The authors developed a generalized microprocessor program for controlling the wheelchair. Zhang et al. [10] merged eye movement information into EEG signal and they used group sparse canonical correlation analysis (GSCCA) for the detection of anxiety emotion. Cheng et al. [11] proposed a method for multi-channel EEG-based emotion recognition using deep forest.

Various research efforts have attempted to create a control mechanism based on EOG signal processing. The method proposed by Xu et al. [12] is a wheelchair control robot system that utilizes the user’s EOG signals to send commands and take control of the wheelchair’s robot. Chowdhury et al. [13] proposed an automated wheelchair based on EOG signal for patients suffering from quadriplegia. The proposed method can control a wheelchair by directional eye movement using an EOG signal. Jambhulkar et al. [14] proposed a system based on the EOG signal for wheelchair control, which can detect the different directions from the user’s eye movements. Mishra et al. [15] proposed a soft wearable electrode system for Parkinson’s disease patients, enabling non-manual control of a wheelchair. A set of electrodes allows users to control the wheelchair by using the EOG data from eye movements. Jagar et al. [16] proposed automatic wheelchair control using eye movement for ALS and Parkinson’s disease patients. The eye-controlled wheelchair contains a web camera for capturing eye movements. The camera is connected to a raspberry pie device that communicates with an Arduino microcontroller to drive the wheelchair. This method uses the pretrained VGG-16 deep network and utilizes transfer learning to improve the network’s accuracy. Barea et al. [17] proposed an automated system for wheelchair control using EOG signal based on neural networks. The system consists of a motorized wheelchair equipped with a mobile computer, sensors placed at the user’s forehead, and a graphical user interface (GUI).

Other research efforts aimed at creating a control mechanism based on hybrid approaches combining EEG and EOG signals. For instance, Huang et al.’s [16] method can control an integrated wheelchair robotic arm system. The user can turn the wheelchair by executing left or right-hand motor imagery. Moreover, he can create other wheelchair or robotic arm commands using eye blinks or eyebrow-raising movements. Bardhan et al. [18] proposed a system for automated wheelchair control, which combines EOG signal captured by Ag/AgCl electrodes and EEG signal captured by a headgear. Both signals are integrated by a microcontroller, which is responsible for controlling the movement of the wheelchair. Bharali et al. [19] proposed an automated wheelchair system for patients with quadriplegia. This system measures the patient’s brain signals using an EEG sensor. The signals are processed and entered as input to an android application. The application is responsible for controlling the wheelchair without the need for manual commands. Finally, the system incorporates EOG signals to enhance its quality.

An essential part of the different systems presented above is the wheelchair user’s EEG and EOG data classification. Some standard machine learning techniques for this task are described below. Naïve Bayes proposed by Maron [20] makes use of Bayes’ theorem and uses probability theory to classify data. This algorithm assumes that all attributes of the data point under consideration are independent of each other. Bayes Network learning utilizes a series of search algorithms and quality measures. It also provides data structures and facilities common to Bayes Network learning algorithms [21]. K-nearest neighbors (K-NN) proposed by Fix and Hodges [22] classifies an object using a plurality vote from its neighbors. The object is classified as the most common class among its k nearest neighbors. The multilayer perceptron (MLP) is another popular classification algorithm. It is a system of interconnected artificial neurons and utilizes a non-linear mapping between a vector containing input data to a vector containing output data [23]. Support vector machine (SVM) is a classifier defined by a separating hyperplane and was initially invented by Vladimir N. Vapnik and Alexey Ya. Chervonenkis in 1963. The SVM algorithm creates an optimal hyperplane, given a training vector containing labeled data, for categorizing new unknown data [24]. The J48-C4.5 decision tree is the WEKA implementation of the decision tree algorithm by Quilan [25]. The decision tree is another popular supervised machine learning classification algorithm where the data are continuously divided according to a specific parameter [25]. Finally, bagging predictors create multiple versions of a predictor, which in turn are used to get an aggregated predictor. In classification problems, the aggregation uses plurality vote to predict a class [26].

This paper proposes an eye recognition system that can capture EEG signals from human subjects using a brain–computer interface (BCI) with the purpose to help patients suffering from severe paralysis, which are unable to use a manual wheelchair. The system utilizes the random forests (RF) classification algorithm to classify the EEG data from the patient’s eye movements into six categories, formed by each corresponding command (a) eyes open, (b) eyes closed, (c) eyes left, (d) eyes right, (e) eyes up, and (f) eyes down. The random forests brain–computer interface (RF-BCI) system aims to be applied in a real-time EEG-based control system for non-manual driving of electromechanical wheelchair rehabilitation devices. The novelty of the proposed system lies on the experimental protocol and particularly on the use of the Emotiv EPOC Flex [27], a lightweight and wearable EEG recording device, with 32 electrodes placed solely on the human’s scalp, for the creation of an EEG dataset, aiming to create a BCI system that will be utilized daily. High levels of accuracy (above 85%) obtained in a 6-class classification problem strengthen the proposed system’s advantages.

## 2. The RF-BCI Architecture

The proposed RF-BCI system uses the EPOC Flex wireless EEG brain wear system equipped with gel Ag/AgCl sensors from Emotiv to record the eye movement data. This system is equipped with 34 sensors, 32 channels for high-density coverage and is easy to use since it can connect and transmit data to a PC at 128 Hz using a wireless connection [27].

The EEG signals were recorded at 128 Hz. Then, with the help of the signal processing tool (sptool, MATLAB 2018a), the unwanted noise was reduced and four 16-order bandpass FIR (Equiripple) digital filter were designed and applied to decompose the EEG recording to specific sub-bands of interest, corresponding to 4 EEG rhythms (delta, theta, alpha, and beta). A high-pass FIR filter with a cut of frequency at 0.5 Hz was also applied to remove low frequencies around 0. Thus, the frequency bands were separated, resulting in the creation of the following four bandpass filters:

- Delta: Has a frequency of 3 Hz or below.
- Theta: Has a frequency between 3.5 and 7.5 Hz.
- Alpha: Has a frequency between 7.5 and 13 Hz.
- Beta: Has a frequency of between 14 and 30 Hz.

Then, the energy in each sub-band of interest was extracted. EEG band energy extraction is a common and straightforward procedure in EEG analysis, revealing much information about the signals recorded and the brain state during the recording. Each rhythm is dominant in a different state. Alpha rhythm is dominant in the posterior region in an EEG recording, while the healthy subject is in a relaxed, seated position with their eyes closed. However, the amplitude and frequency of alpha rhythm attenuates by eye opening and beta rhythm manifests on the presence of a vigilance task (e.g., think left). On the other hand, low rhythms (i.e., delta and theta) are not usually seen during the wakeful EEG of a healthy, adult subject. High levels of energy in these bands would reveal an error in the recording, possible unfiltered artifacts or even a fault in the experimental protocol. Thus, it is crucial to consider and analyze the energy in all four EEG rhythms, according to the following equation:

(1) $$Energy_{i}=\sum_{j=1}^{N}x_{j}{}^{2},\;i\;=\;\delta ,\;\theta ,\;\alpha ,\;\beta ,\;\gamma$$

which is the square value of a data segment $x_{j}$ calculated for each band *i*. Each data segment equals to a 4-second window, that is to say 512 data points for each EEG segment [28].

The EEG features (i.e., band energies) extracted from all the EEG channels provided by the Emotiv Flex and formed the feature vector. Each row of the feature vector corresponds to the recording obtained by one subject and at the end the class attribute is assigned (i.e., “eyes-opened”, ”eyes-closed”, “eyes up”, ”eyes down”,” eyes right”, and ”eyes left”). This process was applied in all 32 channels resulting in 32×4=128 input EEG features for each dataset entry. Then, the channels were further processed, and the sub-band energy was associated with the horizontal and vertical eye movements and the different visual states (open or closed eyes).

**Figure 1:**
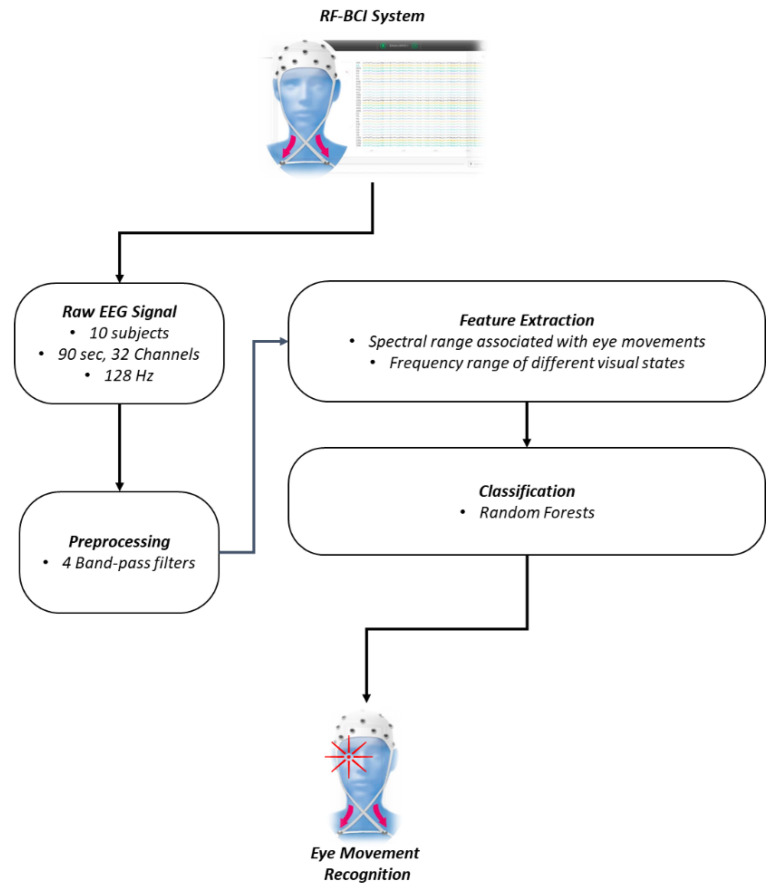
The random forests brain–computer interface (RF-BCI) system architecture. Initially, the EEG signal is captured using the headset and undergoes a preprocessing filtering procedure for removing the unwanted noise and separate the bands. The next step is the feature extraction and the association of each dataset entry with an eye movement. Finally, the dataset is used to train the RF classifier.

The created dataset was divided into training and testing using k-fold cross-validation, where 90% of the samples were used as training data for the RF classifier, and 10% was retained for testing its generalization ability in unknown data. The process is visualized in Figure 1.

## 3. Random Forests

The RF algorithm by Breiman [29] is an extension to the bagging idea [26] and an alternative to boosting. RF has a relatively fast training time and can be applied to regression or classification problems. Other advantages include a small number of user-defined parameters, ease of use in high dimensional problems since they do not need any adaptation, and easy implementation in parallel systems [30].

RF is a tree-based ensemble algorithm with every tree having a dependence on a randomly chosen variable set. Specifically, for a real-valued random vector $X={\left(X_{1},\ldots ,X_{P}\right)}^{T}$ having p dimension and a randomly chosen target variable Y, an unknown joint distribution $P_{XY}\left(X,Y\right)$ is considered. The purpose of this algorithm is to find a function f(X) following the loss function L(Y, f(X)) for the target variable Y, which minimizes the loss function, as seen in Equation (2):

(2) $$E_{XY}\left(L\left(Y,\;f\left(X\right)\right)\right)$$

The subscripts X and Y declare expectation in accordance with X and Y joint distribution while the term L(Y, f(X)) evaluates the relationship between f(X) and Y. If the outcome from this evaluation is that the f(X) values are far away from Y, it applies a penalty to those values. A common form of the loss for classification problems is the zero−one loss shown in Equation (3).

(3) $$L\left(Y,\;f\left(X\right)\right)=\{\begin{array}{l} 0\;\;\;if\;Y=f\left(X\right)\\ 1\;\;\;\;otherwise \end{array}$$

If the set of possible Y values is denoted by 𝓎, minimizing Equation (2) gives the Bayes rule below.

(4) $$f\left(x\right)=\arg max_{y\in 𝓎}P(Y=y|X=x)$$

Ensembles can construct f using a set named “base learners” $h_{1}\left(x\right),\ldots ,h_{j}\left(x\right)$. These base learners are combined with the purpose to give the “ensemble predictor” f(x). The predictor is using the “voting” formula seen below.

(5) $$f\left(x\right)=\arg max_{y\in 𝓎}\overset{J}{\underset{j=1}{\sum }}I\left(y=h_{j}\left(x\right)\right)$$

In this formula, the $j^{th}$ base learner is a tree defined as $h_{j}\left(X,\Theta_{j}\right)$. $\Theta_{J}$ denotes a collection of randomly chosen variables with all $\Theta_{j}$ being independent for j=1,…,J [30]. 

**Figure 2:**
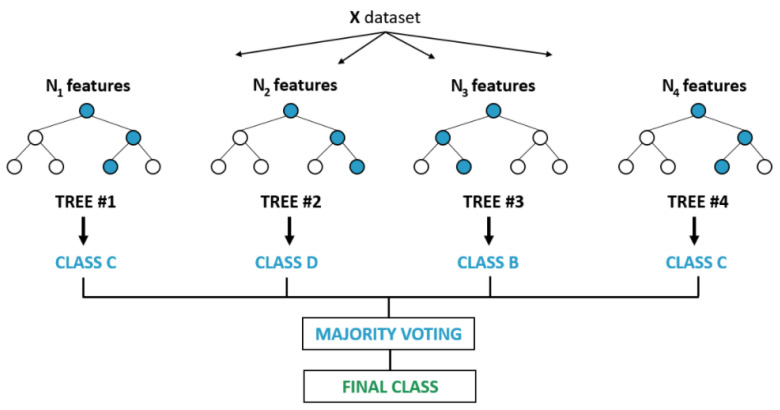
The splitting process on a continuous predictor variable. In the case of a continuous predictor variable, the split is done using a split point. The points where the predictor has a lower value than the split point go to the left. The points with an equal or higher value than the predictor go to the right.

RF uses binary recursive partitioning trees [31,32]. These tree types utilize a series of binary partitions (splits) on individual variables to partition the predictor space. The first element of such a tree is the “root” node, which defines the whole predictor space. The nodes that do not undergo the splitting process are called “terminal nodes” and constitute the final partition. The non-terminal nodes are split into two descendant nodes in compliance with one of the predictor variables’ values. The split is done according to a split point (Figure 2) [30].

Having a set of training data $D=\left\{\left(x_{1},y_{1}\right),\ldots ,\left(x_{N},y_{N}\right)\right\},$ with $x_{i}={\left(x_{i,1},\ldots ,x_{i,p}\right)}^{T}$ indicating the p preditors, $y_{i}$ indicating the response and a specific realization $\theta_{j}$ of $\Theta_{j},$ the fitted tree is defined as $\hat{h_{j}}\left(x,\theta_{j},\;D\right).$ The $\theta_{j}$ component is used to introduce randomness in two ways. The first way is by fitting every tree to an independent bootstrap sample, which is taken from the original data resulting in giving the first part of $\theta_{j}$. The second way is when a node is split, the optimal split is found in a randomly selected subset of m predictor variables instead of using all p predictors. This process is done independently at every node and gives the second part of $\theta_{j}$ [30].

In the original version of the algorithm, Breiman [29] suggested the trees grow until the terminal nodes are pure without using any pruning technique. Segal and Xiao [33] suggested controlling the maximum number of terminal nodes [30]. The outcome from the RF algorithm is extracted by unweighted voting at the resulting trees. The RF algorithm for classification problems can be seen in Algorithm 1 [30].
**Algorithm 1:** The RF Algorithm1: $D=\left\{\left(x_{1},y_{1}\right),\ldots ,\left(x_{N},y_{N}\right)\right\}$ defines the training data with $x_{i}={\left(x_{i,1},\ldots ,x_{i,p}\right)}^{T}.$2: For j=1 to J. 3: Take a bootstrap sample $D_{j}$ of size N from D.4: Fit a tree using $D_{j}$ and binary recursive partitioning.5: Begin with all observations in a single node.6: Repeat for every unsplit node.7: Select m predictors in a random manner from p. 8: Find the optimal binary split on the m predictors.9: Apply the split.10: Until stopping criteria are met.11: To predict a new point *x*, use.$\hat{f}\left(x\right)=\arg max_{y}\sum_{j=1}^{J}I\left(\hat{h_{j}}\left(x\right)=y\right)$ where $\hat{h_{j}}\left(x\right)$. corresponds to the prediction of the response variable at x using the $j^{th}$ tree.

## 4. Simulation Results

The RF-BCI system was tested using a custom created dataset containing data from 10 healthy subjects (5 female and 5 male). The age range was 19–31 for men and 21–28 for women and all participants had a higher level of education. All participants were right-handed and none of them was under medication. Written consent forms to participate in this study were obtained for all the participating subjects.

Experiments were held in a control laboratory environment. During the recording participants were relaxed in an upright, seated position. The records were received wirelessly using the wearable EEG recording device “Emotiv EPOC Flex”. The headset consists of 32 channels, and a specific recording protocol was utilized to capture the eye movements from all samples in a uniform manner. The representation and identification of each user’s eye movements were made using the Emotiv PRO 2 software. The protocol was applied to all ten randomly selected subjects, where seven of them were men and three women. The recordings were repeated four times for each user using the same protocol at each repetition. Each recording’s duration was approximately 90 s, and a baseline, which included six individual steps, was used. Initially, the person was asked to open or close their eyes with a sound indicator. This indicator was used to mark the start and the end of the open and closed eyes. The experimental protocol is based on the following procedure:

- A 3-s preparation time with countdown.
- The eyes remain open for 15 s during the recording.
- A 2-s informational message announcing the completion of the 1st phase.
- A 3-s preparation time with countdown.
- The eyes remain closed for 15 s during the recording.
- A 2-s informational message announcing the completion of the 2nd phase.

After baseline determination, each recording continues with the eyes open, looking towards the center for 10 s. Then, the horizontal and vertical eye movements were recorded in the following order: initially, the user performs the horizontal actions (central to right, right to central, central to left, and left to central) with each movement having a 5-s duration. Then the vertical eye movements (central to upward, upward to central, central to downward, and downward to central) were recoded using the same procedure.

**Figure 3:**
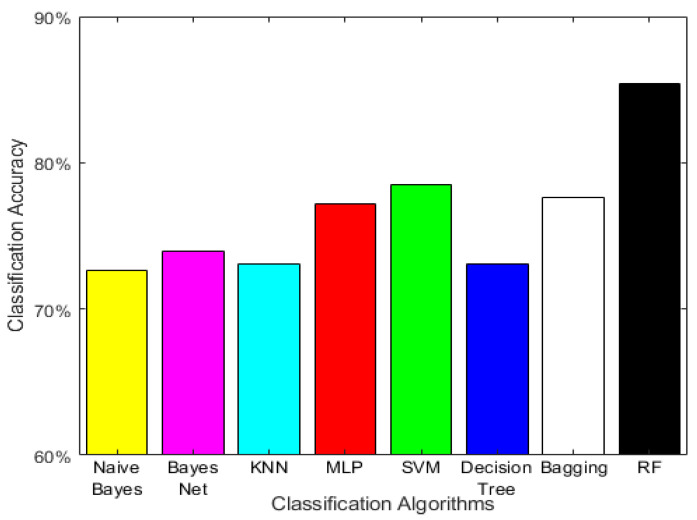
Comparison results bar plot. This bar plot visualizes the test set’s experimental results between the proposed method and seven other popular classification algorithms. It can be seen that the RF method used in RF-BCI produced significantly better results since it was 6.85% better than the 2nd best algorithm (SVM).

After the preprocessing and feature extraction processes were completed, the EEG data taken from the subject’s eye movements were classified into six classes (open, closed, left, right, up, and down) using the RF algorithm. RF was compared using the following seven popular classification algorithms: naïve Bayes, Bayes Network, K-NN, MLP, SVM, J48-C4.5 decision tree, and Bagging. The Waikato environment for knowledge analysis (WEKA) was used to run the above seven classification algorithms using the proposed default values for each algorithm. The dataset was divided into training and test sets using the 10-fold cross-validation method. As seen from the experimental results on the test set in Figure 3, the proposed method has significantly better results (85.39% accuracy) compared to the accuracy achieved from naïve Bayes (72.6%), Bayes Network (73.97%), K-NN (73.06%), MLP (77.17%), SVM (78.54%), J48-C4.5 decision tree (73.06%), and Bagging (77.63%). It should be noted that the 2nd best method (SVM) had a 6.85% decrease in accuracy, which experimentally validates the superiority of the RF algorithm in this dataset over the other five methods.

Table 1 presents the confusion matrix for the random forests classification, which indicated the best classification accuracy. The percentage of classified instances for each class (row) was calculated and depicted in the table. More specifically:

- Class A is formed of the “eyes open” EEG signals.
- Class B is formed of the “eyes closed” EEG signals.
- Class C is formed of the “eyes left” EEG signals.
- Class D is formed of the “eyes right” EEG signals.
- Class E is formed of the “eyes up” EEG signals and
- Class F is formed of the “eyes down” EEG signals.

The main diagonal includes the TP rate of each respective row class, while all other values in the same row are the misclassification rates of this class to all other classes. The confusion matrix that is presented in Table 1, gives a thorough description of the classification.

From Table 1 it can be seen that the best discrimination is provided with signals of class A (96.77%) and class B (95.35%), while the percentage of class C signals correctly classified as class C and the percentage of class D signals correctly classified as class D, show the worse discrimination (78.05% and 75.61% respectively). For signals of class C many instances were misclassified as class D (19.51%) and some of them as class E (2.44%). For signals of class D a significant number of signals are misclassified as class C (24.39%). Additionally, the percentages of correctly classified class E and class F signals were above 80% (83.87% and 84.38% respectively) and the majority of errors was due to misclassified signals as class B (6.45%), class C (3.23%), and class F (6.45%) and class C (3.13%), class D (3.13%), and class E (9.38%) respectively.

## 5. Discussion

In this paper, a BCI-based methodology for 6-class automatic eye movement detection is presented. The EEG signals were acquired from ten volunteers and recorded with the 32-channel Emotiv EPOC Flex. Then, the EEG recordings were analyzed into four bands of interest wherein the main EEG rhythms were found. Finite impulse response (FIR) filters were applied to the EEG signals for extracting the main EEG rhythms. These EEG rhythms were used to train several classifiers, aiming to discriminate the six different types of eye movements, namely “eyes open”, “eyes closed”, “eyes left”, “eyes right”, “eyes up”, and “eyes down”. These commands could be easily translated into “eyes open” and “eyes closed”, and commands “turn left”, “turn right”, and “move forward”, “move backward”, respectively, in an integrated BCI system. RF obtained the best classification accuracy (85.29%).

Intelligent BCI wheelchair systems are of high priority in EEG-based applications, and different approaches have been presented targeting to provide hands-free control and navigation. Several other recent EEG-based studies for real-time wheelchair control systems are presented in Table 2. In all studies, spontaneous EEG was recorded, and no Event-Related Potential (ERP) was provided. The term ERP defines the measured brain response that is the direct result of a specific sensory, cognitive, or motor event. Zgallai et al. [8] and Sim et al. [34] proposed an FFT-based methodology from EEG signals obtained from the Emotiv EPOC+ showing good classification performance. Ben Taher et al. [35] also utilized EEG signals from the Emotiv EPOC+. However, the EPOC+ contains saline-based electrodes that dry fast and are not that stable for long-term recording, compared to the gel-based sensors of EPOC Flex, contaminating EEG with skin artifacts while questioning data quality. Thus, EPOC+ is not a reliable device for long-term recording and is not suitable for a challenging purpose such as non-manual wheelchair control.

Regarding the proposed experimental protocol, the duration of each eye movement was chosen to 5 s only. The extraction of spectral EEG features from this small duration of EEG signal was proven to be enough, showing good discrimination of the six different types of eye movements with an efficient and fast-training machine learning algorithm. We shall note that, in the first-ever developed application of a brain-controlled wheelchair [36], the user was required to imagine the left and right commands for 20 s. The comparison of the proposed methods with previous studies is certainly not straightforward, since different EEG datasets have been utilized and also a different classification problem. The proposed methodology is examined on a multiple classification problem (i.e., 6-class), whereas previous methods have only trained and tested their classifier on binary, 3-class, and 4-class problems. Increasing the number of classes to be discriminated directly affects the classification performance.

Therefore, the current offline approach shows great potential to be applied in a real-time BCI system and paves the way for more research and development on the urgent topic of fully automated EEG-controlled wheelchair systems [38]. Additionally, the use of solely EEG signals and not EOG or a combination of EEG and EOG signals is an advantage of this method. Usually, a headband is more efficient and acceptable from patients than EOG electrodes placed around the eye area that may cause discomfort to the rider, who will not tolerate the entire BCI system [37]. Moreover, in this study, spontaneous EEG was recorded. No ERP or steady-state visual evoked potentials (SSVEP) were provided, allowing the participant to be more independent than pace with system commands. This paper aimed to enhance the experimental protocol with eye blinking and examine the classifier’s performance.

A limitation of the proposed study might be considered as the restricted number of participants. While the sample may seem small, the proposed method shows good classification performance with an accuracy of over 85% for a multiclass classification problem. Emotiv EPOC Flex is a sophisticated EEG recording device with a wearable cap that provides stability and a good, constant sensor connection. Undoubtedly EPOC Flex is more appropriate compared to the former model, EPOC+. However, the device’s setup needs time, which might be a possible drawback for the potential wheelchair rider. Furthermore, regarding feature extraction that is a major step in our EEG methodology, EEG band energy extraction is a common and straightforward procedure in EEG analysis, revealing many information about the signals recorded and the brain state during the recording. The classification accuracy for our proposed 6-class classification problem was above 85% and it was acceptable for a multiclass problem. The recognition accuracy may be improved with the calculation of more spectral and statistical features; however, the addition of more features from 32 channels would require a feature selection method and would definitely increase both the computational burden and the complexity of the proposed method, rendering impossible for our method to be applied in a real-time application.

## 6. Conclusions

In this paper, a BCI method for capturing EEG signals from human subjects using the EPOC Flex wireless EEG brain wear system was presented. After a preprocessing and feature extraction stage, this method was able to classify the EEG recordings into six categories using an RF algorithm. The RF process was chosen because it has a relatively fast training time, a small number of user-defined parameters and superior performance compared to the other five widely used classification algorithms. Our ambition is to apply this method efficiently in a real-time integrated EEG-based electronic wheelchair system for hand-free control and movement and facilitate quadriplegic people who need constant help from a caregiver, increasing their independence and improving the quality of their life.

## Figures and Tables

**Table 1:** Results in terms of accuracy for each eye movement and visual state for random forests.

	Predicted Values					
	Class A	Class B	Class C	Class D	Class E	Class F
class A	96.77	3.23	0	0	0	0
class B	0	95.35	0	0	2.33	2.33
class C	0	0	78.05	19.51	2.44	0
class D	0	0	24.39	75.61	0	0
class E	0	6.45	3.23	0	83.87	6.45
class F	0	0	3.13	3.13	9.38	84.38

**Table 2:** A comparison of the performances of the various methods proposed in the literature for wheelchair navigation.

Study	Control Method	No. of Participants	Device	No. of Channels	Methodology	Classification Accuracy
Zgallai et al. [8]	Mental commands	10	Emotiv EPOC+	14	Fast Fourier Transform (FFT), *θ*, *α*, *β*, *γ*, CNN	96.29%
Sim et al. [34]	Mental commands	5	Emotiv EPOC+	14	FFT, (4-class)	90.00%
Tanaka et al. [36]	Mental commands	6	Not reported	13	Filtering, FFT, standard deviation, correlation coefficient, (2-class)	80.00%
Ben Taher et al. [35]	Eye movement	Not reported	Emotiv EPOC+	14	4-class	Not reported
Aziz et al. [37]	Eye movement	20	g-USBAMP	4	Filtering, *δ*, *α*, variance, central tendency measurement Hidden Markov Model, SVM (3-class)	98.00%
This study	Eye movement	10	Emotiv EPOC Flex	32	Filtering, *δ*, *θ*, *α*, *β*, Random Forests (6-class)	85.39%

## Data Availability

New data were created and analyzed in this study. Data sharing not applicable.
